# TeamSTEPPS Teamwork and Nurses’ Vigilance in Critical Care: A Cross‐Sectional Correlational Study

**DOI:** 10.1155/jonm/7441795

**Published:** 2026-09-29

**Authors:** Amal Diab Ghanem Atalla, Ibrahim Alasqah, Amira M. Ali, Marwa S. Sorour, Nada Alqarawi

**Affiliations:** ^1^ Department of Nursing Administration, Faculty of Nursing, Alexandria University, Alexandria, Egypt, alexu.edu.eg; ^2^ Department of Community, Psychiatric, and Mental Health Nursing, College of Nursing, Qassim University, Buraydah 52571, Saudi Arabia, qu.edu.sa; ^3^ Nursing Services Department, Qassim University Medical City, Buraydah 52571, Saudi Arabia; ^4^ Department of Psychiatric Nursing and Mental Health, Faculty of Nursing, Alexandria University, Alexandria, Egypt, alexu.edu.eg; ^5^ Department of Nursing Administration, Faculty of Nursing, Tanta University, Tanta, Egypt, tanta.edu.eg

**Keywords:** critical care nursing, nurses’ vigilance, patient care, situation monitoring, TeamSTEPPS, teamwork

## Abstract

**Aim:**

To examine the association between perceived TeamSTEPPS teamwork and nurses’ vigilance among critical care nurses and to determine whether overall perceived teamwork statistically predicts nurses’ vigilance.

**Background:**

Vigilance is essential in critical care settings, where nurses must continuously monitor patients and rapidly identify clinical deterioration. Team Strategies and Tools to Enhance Performance and Patient Safety (TeamSTEPPS) is an evidence‐based framework that strengthens teamwork through communication, leadership, situation monitoring, mutual support, and team structure. However, evidence regarding its influence on nurses’ vigilance in critical care environments remains limited.

**Methods:**

We conducted a cross‐sectional descriptive correlational study in accordance with the STROBE guidelines across 24 critical care units at Tanta University Main Hospital. A total of 306 critical care nurses participated. We collected data using a demographic questionnaire, the TeamSTEPPS Teamwork Perceptions Questionnaire (T‐TPQ), and the Nursing Vigilance Scale (NVS). Descriptive statistics, Pearson’s correlation, and simple linear regression analyses were performed using IBM SPSS version 26.0.

**Results:**

Overall TeamSTEPPS teamwork was strongly and positively correlated with nurses’ vigilance (*r* = 0.731, *p* < 0.001). All TeamSTEPPS dimensions demonstrated significant positive associations with vigilance. Among the teamwork dimensions, situation monitoring (*r* = 0.659) and leadership (*r* = 0.648) exhibited the most robust correlations with overall vigilance, while communication recorded the lowest mean percentage score among the teamwork domains.

**Conclusion:**

TeamSTEPPS teamwork was significantly and positively associated with vigilance in critical care settings and was a significant predictor of vigilance scores in the regression model. Due to the cross‐sectional nature of the design, these results should be interpreted as statistical associations rather than causal effects. Since teamwork competencies are important for clinical work, further research is needed to explore causal relationships between teamwork competencies, proactive patient monitoring, and patient safety, including longitudinal and intervention studies.

**Implications for Nursing Management:**

Nursing managers should prioritize strengthening teamwork practices and teamwork‐related competencies to improve communication, mutual support, and situational awareness among critical nurses, thereby enhancing vigilance and overall patient safety outcomes.

## 1. Background

Teamwork is essential in the high‐stakes environment of critical care, where effective communication and coordinated efforts directly impact patient outcomes. The Team Strategies and Tools to Enhance Performance and Patient Safety (TeamSTEPPS) framework comprises 35 items organized into five key dimensions: team structure, leadership, situation monitoring, mutual support, and communication. Each dimension plays a crucial role in the collaborative process, ensuring that critical care nurses can navigate the complexities of patient care efficiently. For example, a well‐defined team structure establishes clear roles and responsibilities, fostering accountability and enhancing performance among team members [1]. In high‐pressure situations typical of critical care settings, an effective leadership style is vital to guide the team, streamline decision‐making, and promote a culture of trust and cooperation [2].

Effective leadership within critical care teams is linked to improved outcomes. Leaders facilitate communication and create an environment where nurses feel empowered to voice concerns and share insights. This empowerment is essential for promoting a culture of safety and can result in significant improvements in patient care quality. Research has shown that critical care units led by strong, transformational leaders have lower rates of adverse events and higher staff satisfaction [3]. Additionally, implementing TeamSTEPPS training can enhance leaders’ skills in managing the dynamic environment of critical care, thereby optimizing team performance and patient safety [4]. The leadership dimension, when effectively executed, ensures that critical care teams can maintain high levels of situation awareness and adaptability, which are critical in times of crisis [5].

Situation monitoring plays a pivotal role in team effectiveness, especially in critical care scenarios where patient conditions can change rapidly. Critical care nurses must be vigilant, continuously assessing both patient status and team dynamics [6]. Training in situation monitoring can lead to improved recognition of patient deterioration and quicker interventions, ultimately improving patient outcomes. Studies show that when teams employ systematic situation monitoring techniques, they can better anticipate problems and enhance the overall quality of care provided [7]. Furthermore, this awareness can facilitate mutual support among team members, promoting a collaborative atmosphere where nurses assist one another in making timely clinical decisions [8].

The communication dimension of TeamSTEPPS is essential to the operational success of critical care nursing teams. Effective communication strategies are crucial for information exchange and ensuring that all team members are on the same page regarding patient care plans [9]. Miscommunication can lead to errors, potentially compromising patient safety. Research indicates that implementing structured communication tools like SBAR (Situation, Background, Assessment, Recommendation) within critical care teams significantly enhances the clarity and efficiency of communication, thereby reducing the likelihood of misunderstandings [10]. Additionally, the use of these structured tools fosters a culture of open dialog, allowing team members to provide and receive feedback constructively [11]. By integrating the principles of TeamSTEPPS into their practice, critical care nurses can cultivate an environment where teamwork thrives, resulting in improved overall patient care and enhanced job satisfaction among team members [12].

Nurses play a pivotal role in promoting patient safety and ensuring quality care in critical care settings, where patients are often in unstable conditions requiring constant monitoring. Vigilance in nursing refers to the ability to maintain vigilance and awareness of patient statuses, as well as to recognize subtle changes in clinical conditions that may indicate deterioration [13]. Critical care nurses are trained to utilize various assessment tools, clinical guidelines, and evidence‐based practices to enhance their vigilance and decision‐making capabilities. This continuous observation is essential in a critical care environment, where the consequences of missed observations or delayed interventions can be dire. Moreover, research indicates that high levels of vigilance among nurses correlate with improved patient outcomes, including reduced rates of adverse events and lower mortality rates [14].

The concept of vigilance extends beyond individual nursing assessments; it is also a team‐based approach that requires effective communication and collaboration among all healthcare professionals involved in a patient’s care [15]. In high‐risk environments, such as intensive care units (ICUs), teamwork and situational awareness are critical for maintaining optimal patient safety. For instance, implementing structured communication frameworks, like TeamSTEPPS, enhances collaboration among nurses, physicians, and other healthcare providers, thereby improving vigilance [16]. Research shows that a shared mental model and mutual support among team members enhances the overall level of vigilance, allowing for timely interventions that can potentially save lives [17]. Additionally, ongoing education and training in situation awareness and monitoring techniques are vital, as they equip nurses with the skills needed to respond to rapidly changing patient conditions effectively [18].

Despite the challenges posed by high patient acuity and complex care demands, fostering a culture of vigilance among critical care nurses is imperative. Strategies to enhance vigilance include promoting a culture of safety where nurses feel empowered to speak up about concerns, providing adequate staffing levels to prevent burnout, and utilizing technology to support monitoring efforts [19]. Research has shown that a positive work environment that encourages vigilance can reduce the likelihood of errors and improve overall patient satisfaction [20]. Furthermore, a systematic approach to monitoring that incorporates both human and technological resources can lead to more proactive nursing care and enhanced patient safety. As such, priority should be given to interventions that support and sustain vigilance among critical care nurses, recognizing their indispensable role in patient care and safety [21].

### 1.1. Theoretical Framework

Teamwork theory emphasizes the importance of collaboration and effective communication among team members. In high‐stakes environments like critical care units, nurse vigilance can be enhanced through established teamwork structures that promote clear roles, accountability, and mutual support [22]. Teamwork models, such as the input–process–output model, illustrate how teamwork processes, such as shared mental models and collaborative problem‐solving, can lead to improved outcomes, including enhanced vigilance in monitoring patient conditions. When critical care nurses engage in collaborative practices, they can effectively distribute monitoring tasks, share information, and respond collectively to potential patient deterioration [23].

### 1.2. Significance of the Study

This study aims to improve patient safety and care quality in critical care settings by examining how nurses’ perceptions of teamwork are associated with nurses’ vigilance. Given that critical care nurses work in high‐pressure environments where timely interventions are essential, understanding the role of structured teamwork can inform nursing education and clinical practices. By identifying how teamwork enhances situational awareness and fosters a culture of safety, the research may lead to strategies that improve collaboration and reduce adverse events, ultimately benefiting patient recovery. Additionally, the findings could guide healthcare organizations in effectively implementing TeamSTEPPS, influencing policy and resource allocation to promote proactive nursing teams.

### 1.3. Research Questions

- What is the relationship between TeamSTEPPS teamwork and nurses’ vigilance among critical care nurses?
- What is the predictive effect of overall TeamSTEPPS teamwork on nurses’ vigilance among critical care nurses?

### 1.4. Research Hypotheses

Based on the theoretical framework, study objectives, and evidence from previous research, the following hypotheses were formulated:

 H1: Higher perceived TeamSTEPPS teamwork is significantly and positively associated with higher levels of nurses’ vigilance in critical care settings.
 H2: Higher scores on the individual dimensions of TeamSTEPPS teamwork, including team structure, team leadership, situation monitoring, mutual support, and communication, are significantly and positively associated with nurses’ vigilance.

## 2. Method

### 2.1. Study Design and Participants

We used a cross‐sectional descriptive correlational design based on Strengthening the Reporting of Observational Studies in Epidemiology (STROBE) guidelines. The target population consisted of 365 nurses in the participating critical care units. Of these, 31 nurses were included in the pilot study and were excluded from the main study. Among the rest of the nurses, 12 nurses were excluded due to less than 6 months of employment. Finally, 322 nurses were eligible. Of the 322 eligible nurses, 16 refused to participate, and 306 completed the study questionnaires and were included in the final analytic sample. Thus, the response rate among eligible nurses was 95.0% (306/322) (Supporting Figure 1).

### 2.2. Sample Size and Sampling Technique

The required sample size was calculated using Cochran’s formula for cross‐sectional studies at a 90% confidence level and 5% margin of error. A 90% confidence level was selected to achieve a reasonable balance between statistical precision and feasibility, given the practical constraints of data collection across 23 critical care units and the available target population. This approach was considered appropriate for the cross‐sectional nature of the study while facilitating adequate recruitment across the participating critical care units

(1) n=Z2P1−Pd2,

where

• *n* = required sample size
• *Z* = standard normal deviation corresponding to the 90% confidence level (1.645)
• *P* = estimated proportion of the target population. A conservative estimate of 50% (*P* = 0.50) was used due to the absence of previous local studies
• *d* = margin of error set at 5% (0.05)

Substituting the values into the equation:

(2) n=1.6452×0.5010.50−0.052=270.6271≈.

To compensate for potential nonresponse and incomplete questionnaires, a 13% contingency allowance was added:

(3) nadjusted=2711.13×,nadjusted=306.2306≈.

This study was carried out at Tanta University’s Main University Hospital. With a capacity of almost 6760 beds, it offers public health services that are not paid for. It is Tanta’s biggest university hospital for education. For descriptive and comparative analyses, the 24 critical care units were aggregated into broader specialty categories based on the similarity of clinical services and patient populations. This grouping was performed to facilitate interpretation of demographic characteristics and to ensure adequate numbers of participants within each category. The aggregated categories reported in Table 1, therefore, represent combinations of individual critical care units rather than separate hospital departments.

**TABLE 1:** Distribution of nurses studied according to demographic characteristics.

Demographic characteristics	No.	%
*Age*		
≤ 30 years	33	10.8
31 ≤ 50 years	205	67.0
≥ 51 years	68	22.2
Mean ± SD	44.8 ± 9.50	

*Gender*		
Female	306	100.0
Male	0	0.0

*Residence*		
Rural	94	30.7
Urban	212	69.3

*Level of education*		
Technical Nursing institute	119	38.9
Bachelor in Science of nursing	123	40.2
Post graduate studies	64	20.9

*Years of experience*		
< 4	199	65.0
4–8	79	25.8
> 8	28	9.2
Mean ± SD	5.10 ± 2.1	

*Department*		
Cardiac care unit	46	15.0
Psychiatry and Neurology care unit	151	49.3
Pediatric intensive care unit	53	17.3
Neonatal intensive care unit	38	12.4
Medical intensive care unit	8	2.6
Chest intensive care unit	10	3.3

All 24 critical care units were included in the study: the Second Unit, Third Unit, Fourth Unit, Medical Emergency Unit, Surgical Emergency Unit, Intensive Care Unit of Emergency Operation, Transitional Intensive Care Unit, New Transitional Unit, Continuous Renal Replacement Therapy, Critical Cases, Intensive Care Unit, Toxicity Unit, Maxillofacial and Plastic Surgery ICU, Burn ICU, Pulmonology ICU, Neurosurgery ICU (1), Neurosurgery Pediatric ICU, Hematemesis ICU, Urology ICU, Anesthesia ICU, Systemic Lupus ICU, Hepatic Transitional ICU, Diabetes ICU, and Ear, Nose, and Throat ICU (ENT).

For the descriptive and comparative analyses, the 24 critical care units were pooled into broader specialty categories based on the similarity of clinical services and patient populations. This grouping was done to aid interpretation of demographic characteristics and to ensure adequate numbers of participants within each category. Therefore, the combined categories reported in Table 1 are made up of individual critical care units and not independent hospital departments. For the specific mapping of each critical care unit to its corresponding aggregated specialty category, please see Supporting Table 1. It has six aggregated categories, namely, Cardiac, Psychiatry and Neurology, Pediatric, Neonatal, Medical, and Chest, with 306 nurses distributed among them.

### 2.3. Procedure for Data Collection

The competent authorities of the chosen critical care units at the Main University Hospital, Tanta University, provided formal written consent to perform the study before the start of data collection. After that, the researchers went to the study locations in person and gave the surveys to the qualified critical care nurses during their shifts at convenient times that did not conflict with patient care tasks. Each participant met with the researchers one‐on‐one to discuss the significance and goal of the study, give information on how to complete the questionnaires, and extend an invitation to voluntarily participate. The Nursing Vigilance Scale (NVS), the TeamSTEPPS Teamwork Perceptions Questionnaire (T‐TPQ), and the demographic data sheet were among the research tools. It took about 20–25 min to complete the questionnaires. To give participants enough time for data collection and follow‐up, the fieldwork phase was conducted over 3 months, from November 2025 to February 2026.

### 2.4. Study Instruments

Two instruments were used in this study.

#### 2.4.1. Tool (1): T‐TPQ

The T‐TPQ was developed as part of the TeamSTEPPS program by the Agency for Healthcare Research and Quality (AHRQ) in collaboration with the U.S. The questionnaire was translated into Arabic using a forward–backward translation methodology and culturally adapted for use among Arabic‐speaking critical care nurses while preserving the original items, response options, and scoring procedures. The 35 items are divided into five categories: Communication (7 items), Team Structure (7 items), Leadership (7 items), Situation Monitoring (7 items), and Mutual Support (7 items). A 5‐point Likert scale, from strongly disagree (1) to strongly agree (5), was used to gauge responses. The total score ranges from 35 to 175. For descriptive purposes only, total teamwork scores were classified into three categories (low, moderate, and high) by dividing the theoretical score range into approximately equal intervals. These categories were not based on validated clinical or normative cutoff scores provided by the original instrument developers and have not been validated among Arabic‐speaking critical care nurses. Continuous scores were retained for all inferential statistical analyses.

Higher scores indicate a higher perceived level of teamwork. Cronbach’s alpha ranged from 0.88 to 0.94 in earlier research, indicating outstanding reliability. Cronbach’s alpha reliability was 0.94 in this study.

T‐TPQ’s internal consistency reliability was excellent in the initial investigation, with Cronbach’s alpha coefficients for each of the five dimensions ranging from 0.88 to 0.95. Furthermore, an overall Cronbach’s alpha of 0.942 was obtained in the Iranian validation study by Hojat et al. (2025) [24], indicating excellent reliability.

A demographic data sheet was used to collect information on participants’ age, gender, marital status, educational background, work unit, and years of professional experience.

#### 2.4.2. Tool (2): NVS

Ajri‐Khameslou et al. (2021) [25] used this instrument, which was created, to evaluate nurses’ vigilance in critical care practice. The scale was translated into Arabic using a forward–backward translation methodology and culturally adapted for use among Arabic‐speaking critical care nurses while preserving the original items, response options, and scoring procedures. The 40 items are divided into three categories: Clinical Decision‐Making (17 items), Knowing the Patient for Pattern Recognition (13 items), and Timely Identification of Patient Change (10 items). A 5‐point Likert scale, from never (1) to always (5), was used to gauge the responses. The overall score ranges from 40 to 200. For descriptive presentation only, total vigilance scores were divided into low, moderate, and high categories using approximately equal divisions of the theoretical score range. These categories were not derived from validated clinical thresholds and were not used in the primary inferential analyses. Higher scores are indicative of more vigilant nurses. The NVS showed acceptable internal consistency, with an overall Cronbach’s alpha of 0.74 [25]. According to the current study, Cronbach’s alpha reliability was 0.88.

### 2.5. Ethical Considerations

The Research Ethics Committee of the Tanta University’s Faculty of Nursing granted ethical permission for the study. Before the start of data collection, official permission to conduct the study was also acquired from the relevant hospital authorities and the Dean of Tanta University’s Faculty of Nursing (approved No. 964/5/2025). Individual participant consent was neither replaced nor constituted by the hospital administration’s institutional authorization to enter the study setting and recruit qualified nurses.

No observational component was conducted as part of the present study. Data were collected exclusively through self‐administered questionnaires. All eligible nurses were informed about the study purpose, procedures, voluntary nature of participation, and their right to decline participation or withdraw without any consequences. Written informed consent was obtained from each participating nurse before questionnaire completion.

Participant autonomy, privacy, and confidentiality were preserved throughout the study. Participation was completely voluntary, and participants were not subjected to any penalty or disadvantage for declining or withdrawing. Questionnaires were completed anonymously, and no personally identifying information was included in the study dataset. Data were handled confidentially and used solely for research purposes. Standardized data‐collection procedures were applied across the participating critical care units to minimize potential informational and social desirability biases.

### 2.6. Validity

Before the study instruments were used and translated, explicit permission was received from the original authors/developers. A panel of seven experts from Alexandria University was constituted to guarantee the validity of the instruments used in the current investigation. Experts in nursing administration, critical care nursing, nursing research, and bilingual professionals who were conversant in both Arabic and English, as well as the target population’s cultural background, comprised the panel. The first step in the translation process was forward translation, where two skilled bilingual translators who were familiar with medical jargon independently translated the T‐TPQ and NVS from English into Arabic. The purpose of this stage was to guarantee language clarity and maintain conceptual meaning.

Two independent translators who had not taken part in the forward translation procedure then carried out the backward translation. To find inconsistencies and verify equivalency with the original instruments, the Arabic translations were translated back into English.

To assess face validity (clarity, readability, and comprehensibility) and content validity (relevance, appropriateness, and representativeness of items), the expert panel then examined the original, forward‐translated, and backward‐translated versions. The necessary phrase and phrasing changes were made in accordance with the panel’s suggestions to enhance clarity and cultural appropriateness.

The final reconciled Arabic versions of the measures were then examined to make sure they were appropriate for evaluating TeamSTEPPS teamwork and nurses’ vigilance among critical care nurses and that they were consistent with the original scales.

### 2.7. Pilot Study

Thirty‐one critical care nurses, or 10% of the entire study sample, participated in a pilot study to evaluate the feasibility, application, and clarity of the study instruments, which were the NVS and the T‐TPQ. The pilot study also sought to assess the preliminary validity and reliability of the instruments, estimate the time needed to complete the questionnaires, and identify any potential challenges that might occur during the real data collection procedure. The main study sample did not include participants from the pilot study. According to the pilot research’s findings, no changes were necessary because the study instruments were comprehensible, clear, and suitable for the intended audience. Internal consistency reliability was assessed for each subscale and the total scale using Cronbach’s alpha coefficients. The obtained coefficients demonstrated satisfactory reliability for all dimensions.

### 2.8. Statistical Analysis

Data were coded, entered, and analyzed using IBM SPSS Statistics version 26.0. Data were checked for completeness and accuracy, and the normality of the distribution of quantitative variables was assessed using the Kolmogorov–Smirnov test. Internal consistency of T‐TPQ and NVS was assessed by calculating Cronbach’s alpha coefficient. To examine differences in continuous teamwork and vigilance scores by demographic and occupational characteristics, independent‐samples *t*‐tests and one‐way analysis of variance (ANOVA) were conducted. Pearson’s correlation coefficient was used to analyze the direction and strength of the association between the overall TeamSTEPPS teamwork and nurses’ vigilance and between the respective subdimensions.

Then, a simple linear regression analysis was conducted to explore whether the overall perceived TeamSTEPPS teamwork significantly predicted nurses’ vigilance. Continuous scores were used for correlation and regression analysis. In view of the cross‐sectional study design, the regression model was interpreted as an estimate of statistical prediction and association rather than evidence of a causal effect. Statistical significance was set at *p* < 0.05, and all tests were two‐sided.

## 3. Results

With a mean age of 44.8 years, the sample comprised 306 female nurses, the majority of whom were between the ages of 31 and 50 (67%). Nearly 70% of them lived in cities, and the majority had a bachelor’s degree (40.2%) or a technical nursing diploma (38.9%). Interestingly, two‐thirds had less than 4 years of experience, suggesting that the workforce was primarily in the early stages of their careers. Nurses were spread throughout several specialist areas, with neurology and psychiatry having the largest proportion (49.3%). Given that age, experience, and specialist units subsequently exhibit strong relationships with the research variables, these demographics offer crucial contextual elements when evaluating teamwork and vigilance ratings (see Tables 2–5).

**TABLE 2:** Distribution of the nurses studied according to their levels and mean percent score.

Tools	Low		Moderate		High		Total score	Mean score	Mean percent score
	No.	%	No.	%	No.	%	Mean ± SD	Mean ± SD	Mean ± SD
Nurses’ Vigilance tool	2	0.7	223	72.9	81	26.5	132.36 ± 17.87	3.33 ± 0.45	58.27 ± 11.32
Timely Identification of Patient Change (TIPC)	3	1.0	198	64.7	105	34.3	33.98 ± 5.86	3.40 ± 0.59	59.96 ± 14.64
Knowing the Patient for Pattern Recognition (KPPR)	4	1.3	199	65.0	103	33.7	44.50 ± 7.40	3.42 ± 0.57	60.58 ± 14.22
Clinical Decision Making (CDM)	15	4.9	238	77.8	53	17.3	53.88 ± 8.44	3.17 ± 0.50	54.28 ± 12.42
TeamSTEPPS Teamwork tool	67	21.9	209	68.3	30	9.8	99.68 ± 23.97	2.85 ± 0.68	46.20 ± 17.11
Team structure	84	27.5	176	57.5	46	15.0	19.73 ± 5.51	2.82 ± 0.79	45.46 ± 19.63
Team Leadership	61	19.9	197	64.4	48	15.7	20.67 ± 5.20	2.95 ± 0.74	48.82 ± 18.56
Situation monitoring	67	21.9	192	62.7	47	15.4	20.07 ± 5.58	2.87 ± 0.80	46.66 ± 19.92
Mutual support	78	25.5	169	55.2	59	19.3	20.32 ± 6.04	2.90 ± 0.86	47.57 ± 21.57
Communication	104	34.0	165	53.9	37	12.1	18.90 ± 6.07	2.70 ± 0.87	42.48 ± 21.69

**TABLE 3:** Correlation between the studied variables.

		TIPC	KPPR	CDM	Overall nurses’ vigilance	Team structure	Team leadership	Situation monitoring	Mutual support	Communication	Overall STEPPS
TIPC	*r*										
	*p*										

KPPR	*r*	0.561^∗^									
	*p*	< 0.001^∗^									

CDM	*r*	0.428^∗^	0.545^∗^								
	*p*	< 0.001^∗^	< 0.001^∗^								

Overall nurses’ vigilance	*r*	0.762^∗^	0.855^∗^	0.838^∗^							
	*p*	< 0.001^∗^	< 0.001^∗^	< 0.001^∗^							

Team structure	*r*	0.411^∗^	0.434^∗^	0.572^∗^	0.585^∗^						
	*p*	< 0.001^∗^	< 0.001^∗^	< 0.001^∗^	< 0.001^∗^						

Team leadership	*r*	0.461^∗^	0.525^∗^	0.591^∗^	0.648^∗^	0.644^∗^					
	*p*	< 0.001^∗^	< 0.001^∗^	< 0.001^∗^	< 0.001^∗^	< 0.001^∗^					

Situation monitoring	*r*	0.484^∗^	0.488^∗^	0.631^∗^	0.659^∗^	0.633^∗^	0.685^∗^				
	*p*	< 0.001^∗^	< 0.001^∗^	< 0.001^∗^	< 0.001^∗^	< 0.001^∗^	< 0.001^∗^				

Mutual support	*r*	0.433^∗^	0.481^∗^	0.557^∗^	0.604^∗^	0.593^∗^	0.620^∗^	0.661^∗^			
	*p*	< 0.001^∗^	< 0.001^∗^	< 0.001^∗^	< 0.001^∗^	< 0.001^∗^	< 0.001^∗^	< 0.001^∗^			

Communication	*r*	0.431^∗^	0.454^∗^	0.561^∗^	0.594^∗^	0.617^∗^	0.610^∗^	0.628^∗^	0.710^∗^		
	*p*	< 0.001^∗^	< 0.001^∗^	< 0.001^∗^	< 0.001^∗^	< 0.001^∗^	< 0.001^∗^	< 0.001^∗^	< 0.001^∗^		

Overall STEPPS	*r*	0.526^∗^	0.563^∗^	0.689^∗^	0.731^∗^	0.822^∗^	0.835^∗^	0.852^∗^	0.856^∗^	0.852^∗^	
	*p*	< 0.001^∗^	< 0.001^∗^	< 0.001^∗^	< 0.001^∗^	< 0.001^∗^	< 0.001^∗^	< 0.001^∗^	< 0.001^∗^	< 0.001^∗^	

*Note: r*, Pearson coefficient.

^∗^Statistically significant at *p* ≤ 0.05.

**TABLE 4:** Simple linear regression model analysis examining the association between perceived TeamSTEPPS and self‐reported nursing vigilance.

Factors	*B*	Beta	*t*	*p*	95% CI	
					LL	UL
TeamSTEPPS Teamwork	0.545	0.731	16.672^∗^	< 0.001^∗^	0.487	0.602
*R* ^2^ ** = 0.534, Adjusted** *R* ^2^ ** = 0.533, *F* = 348.654** ^**∗**^ **,** **p** < 0.001^∗^						

*F*, *p*, F, and *p* values for the model; *R* ^2^, coefficient of determination; *B*, unstandardized coefficients; beta, standardized coefficients; *t*, *t*‐test of significance.

Abbreviations: CI, confidence interval; LL, lower limit; UL, upper limit.

^∗^Statistically significant at *p* ≤ 0.05.

**TABLE 5:** Comparison of mean TeamSTEPPS teamwork and nurses’ vigilance scores according to demographic and occupational characteristics.

Demographic characteristics	Nurses’ vigilance						TeamSTEPPS teamwork					
	Low		Moderate		High		Low		Moderate		High	
	No.	%	No.	%	No.	%	No.	%	No.	%	No.	%
*Age*												
≤ 30 years	0	0.0%	28	12.6%	5	6.2%	11	16.4%	22	10.5%	0	0.0%
31 ≤ 50 years	2	100.0%	163	73.1%	40	49.4%	54	80.6%	142	67.9%	9	30.0%
≥ 51 years	0	0.0%	32	14.3%	36	44.4%	2	3.0%	45	21.5%	21	70.0%

**χ** ^2^ (**p**)	**29.215** ^**∗**^ **(**< 0.001^∗^ **)**						**51.009** ^**∗**^ **(**< 0.001^∗^ **)**					

*Residence*												
Rural	1	50.0%	64	28.7%	29	35.8%	15	22.4%	67	32.1%	12	40.0%
Urban	1	50.0%	159	71.3%	52	64.2%	52	77.6%	142	67.9%	18	60.0%

**χ** ^2^ (**p**)	**2.159 (0.296)**						**3.622 (0.178)**					

*Level of education*												
Technical Nursing institute	1	50.0%	83	37.2%	35	43.2%	20	29.9%	91	43.5%	8	26.7%
Bachelor in Science of nursing	0	0.0%	92	41.3%	31	38.3%	36	53.7%	74	35.4%	13	43.3%
Postgraduate studies	1	50.0%	48	21.5%	15	18.5%	11	16.4%	44	21.1%	9	30.0%

**χ** ^2^ (**p**)	**2.903 (0.574)**						**9.876** ^**∗**^ **(0.043** ^**∗**^ **)**					

*Years of experience*												
< 4	1	50.0%	169	75.8%	29	35.8%	67	100.0%	132	63.2%	0	0.0%
4–8	1	50.0%	45	20.2%	33	40.7%	0	0.0%	68	32.5%	11	36.7%
> 8	0	0.0%	9	4.0%	19	23.5%	0	0.0%	9	4.3%	19	63.3%

**χ** ^2^ (**p**)	**47.288** ^**∗**^ **(**< 0.001^∗^ **)**						**138.107** ^**∗**^ **(**< 0.001^∗^ **)**					

*Department*												
Cardiac care unit	0	0.0%	29	13.0%	17	21.0%	7	10.4%	37	17.7%	2	6.7%
Psychiatry and Neurology care unit	1	50.0%	118	52.9%	32	39.5%	31	46.3%	108	51.7%	12	40.0%
Pediatric intensive care unit	1	50.0%	34	15.2%	18	22.2%	11	16.4%	31	14.8%	11	36.7%
Neonatal intensive care unit	0	0.0%	29	13.0%	9	11.1%	12	17.9%	23	11.0%	3	10.0%
Medical intensive care unit	0	0.0%	6	2.7%	2	2.5%	3	4.5%	4	1.9%	1	3.3%
Chest intensive care unit	0	0.0%	7	3.1%	3	3.7%	3	4.5%	6	2.9%	1	3.3%

**χ** ^2^(**p**)	**11.191 (0.366)**						**14.607 (0.108)**					

*Note:* **χ** ^2^, chi‐squared test. ^∗^Statistically significant at *p* ≤ 0.05.

Abbreviation: MC, Monte Carlo.

The findings show that over one‐quarter of nurses achieved high vigilance (26.5%), while the majority reported moderate levels of vigilance (72.9%). Subdimensions revealed comparable trends: CDM had fewer nurses in the high range (17.3%), indicating that decision‐making vigilance is comparatively lower, whereas TIPC and KPPR had almost one‐third of nurses in the high category. With only 9.8% at strong teamwork and 68.3% at moderate teamwork, TeamSTEPPS results were generally lower. Leadership and situation monitoring fared relatively better than communication, which had the lowest mean percent score among the cooperation subdimensions, indicating a crucial area that requires improvement. The study’s hypothesis that cooperation quality varies across dimensions and may affect vigilance levels is supported by these patterns.

Overall TeamSTEPPS demonstrated a strong positive correlation with overall nurses’ vigilance (*r* = 0.731, *p* < 0.001). Furthermore, all TeamSTEPPS dimensions showed statistically significant positive correlations with total vigilance and its three dimensions (Clinical Decision‐Making, Knowing the Patient for Pattern Recognition, and Timely Identification of Patient Change). Communication and mutual support exhibited particularly strong associations with overall vigilance, while situation monitoring showed the strongest relationship with clinical decision‐making vigilance. These findings suggest that multiple teamwork competencies are associated with enhanced vigilance behaviors among critical care nurses. Correlations between overall TeamSTEPPS scores and their component dimensions were not interpreted because they represent part–whole relationships.

A simple linear regression analysis demonstrated a statistically significant association between overall perceived TeamSTEPPS teamwork and nurses’ vigilance. Overall TeamSTEPPS teamwork significantly predicted vigilance scores (*β* = 0.731, *p* < 0.001), with the model explaining 53.3% of the observed variance in vigilance scores (adjusted *R* ^2^ = 0.533). The 95% confidence interval for the regression coefficient was 0.487–0.602. These findings indicate a substantial statistical association between perceived teamwork and vigilance; however, the cross‐sectional design does not permit causal inferences.

Analysis of mean vigilance and teamwork scores across demographic and occupational groups showed significant differences according to age and years of experience. Older and more experienced nurses demonstrated significantly higher teamwork and vigilance scores than their younger and less experienced counterparts. Educational level was significantly associated with teamwork scores, whereas differences in vigilance scores were not statistically significant. No significant differences were observed according to critical care unit type.

## 4. Discussion

As the demand for high‐quality care in critical settings continues to rise, ensuring patient safety and enhancing nursing effectiveness have become paramount. Critical care nurses operate in complex, high‐stake environments where lapses in vigilance can lead to severe consequences for patient outcomes (Z. Zhang et al., 2026) [26]. The results of this study emphasize the importance of teamwork in relation to nurses’ vigilance in critical care settings. The work environments of critical care nurses are complex, high‐risk environments that require continuous patient monitoring, timely recognition of clinical changes, and coordinated responses. The present study demonstrated that higher levels of nurses’ vigilance were strongly associated with higher perceived TeamSTEPPS teamwork. These findings underscore the potential importance of structured teamwork competencies, especially communication, leadership, situation monitoring, and mutual support, for effective patient monitoring and safety. However, as the study was cross‐sectional, the observed associations should not be interpreted as evidence of a causal effect of teamwork on higher vigilance.

The findings of the study highlight a multifaceted landscape of vigilance among critical care nurses, revealing that while a small proportion of nurses achieve high levels of vigilance, the majority fall into moderate categories. This moderate level of vigilance could be indicative of various systemic and contextual factors affecting nursing practices. The findings of this study (Birkholz et al., 2025) [27] reveal a nuanced picture of vigilance among critical care nurses, with only 26.5% demonstrating high levels of vigilance, while the majority (72.9%) fall into moderate categories.

This trend may reflect the complexities inherent in critical care environments, where high stress and overwhelming workloads can significantly impede sustained attention and vigilance (Kinnunen et al., 2025) [28]. Notably, the analysis also indicates a concerningly low rate (17.3%) of nurses achieving high decision‐making vigilance, suggesting that factors such as cognitive overload and time constraints contribute to diminished critical thinking abilities in high‐pressure situations (G.‐H. Lin et al., 2025) [29]. Additionally, the limited manifestation of effective teamwork, evidenced by only 9.8% exhibiting strong collaborative dynamics, highlights systemic issues that may impede effective communication, which is foundational to both teamwork and vigilance (van Esch et al., 2026), [30].

Comparatively higher scores in leadership and situation monitoring can be viewed as indicators of effective nursing practices; however, the overall low scores across teamwork dimensions warrant urgent attention to improving collaborative practices essential for fostering vigilance. Factors such as a high‐stress work environment and inadequate training on teamwork and communication may significantly affect these outcomes.

Communication had the lowest mean percentage score of TeamSTEPPS teamwork dimensions (42.48%), which was an unexpected finding. Although the present study did not directly measure the underlying reasons for this finding, communication challenges may be especially relevant in critical care settings characterized by complex processes of care, high workload, time‐sensitive decision‐making, frequent interruptions, and interactions between professionals with different roles and levels of authority. These contextual factors can impede the timely and effective sharing of information even when structured teamwork frameworks are in place. This finding suggests that more research is needed to explore the specific organizational, interpersonal, and contextual barriers that may influence communication among critical care nurses.

Another important finding was that there were no statistically significant differences between perceived TeamSTEPPS teamwork and nurses’ vigilance in different types of critical care units. Differences in patient populations and clinical demands exist across specialties, but this finding may also reflect the impact of factors beyond clinical specialty, such as organizational climate, managerial practices, team functioning, and common workplace processes, on teamwork and vigilance. However, this interpretation needs to be taken with caution because these factors were not directly addressed in the current study. Future work could explore whether organizational and team‐level factors account for variations in teamwork and vigilance across different types of critical care settings Wilson et al., 2024; Kim et al., 2026 [31, 32].

Furthermore, institutional cultures that fail to prioritize open communication can exacerbate deficiencies in teamwork efficacy, ultimately impacting patient safety (Najafi et al., 2026) [33]. Thus, fostering a culture that emphasizes teamwork and communication might be crucial for enhancing vigilance levels in critical care nursing and improving overall patient care quality.

The NVS demonstrated greater internal consistency in the current study (Cronbach’s *α* = 0.88) compared with the original validation study (*α* = 0.74). This difference may be attributable to characteristics of the present critical care nursing sample or to contextual and cultural differences in the interpretation and practice of vigilance. However, the higher alpha coefficient should not be taken as evidence of the better psychometric performance of the Arabic version, as internal consistency may vary across samples and settings. Further psychometric testing of the Arabic version across different nursing populations and healthcare settings is required to confirm the stability and generalizability of its measurement properties.

The study found a significant positive correlation (*r* = 0.731, *p* < 0.001) between nurses’ vigilance and their overall perception of TeamSTEPPS collaboration. Vigilance scores, which include increased vigilance, awareness of patient changes, and clinical decision‐making vigilance, were linked to higher teamwork scores. Significant correlations were also found between vigilance dimensions and communication, mutual support, and situation monitoring. These results imply that nurses’ vigilance in critical care environments may be related to a variety of collaboration qualities. However, the cross‐sectional design makes it impossible to prove whether increased cooperation leads to increased vigilance or to ascertain the directionality of these associations. Najafi et al., 2026; Pineda et al., 2026 [33, 34] further illustrate that various teamwork skills collectively contribute to nurses’ ability to detect patient changes and patterns effectively.

Overall perceived TeamSTEPPS teamwork was found to be a statistically significant predictor of nurses’ vigilance, explaining 53.3% of the observed variance in vigilance levels (*β* = 0.731, *p* < 0.001), according to the regression analysis. This result suggests that, in the current sample, perceived teamwork has a significant statistical explanatory value. However, because the data were gathered at a particular point in time, the regression model should not be taken as showing a causal relationship between cooperation and vigilance. To ascertain if gains in cooperation subsequently result in quantifiable changes in nurses’ vigilance, longitudinal and intervention‐based research is required.

Several mechanisms may underlie these results. Effective communication, a core element of TeamSTEPPS, facilitates clearer information exchange, enhancing mutual support and coordination among team members (Liu et al., 2026) [35]. This improved communication potentially leads to better situation monitoring, enabling nurses to identify subtle yet critical patient condition changes (Aydogdu, 2024) [36]. Additionally, the development of shared mental models fosters anticipation of teammates’ actions, ultimately supporting decision‐making processes. Furthermore, Esmaeili et al., 2026 [37] a cohesive team environment can reduce stress, allowing nurses to concentrate more on patient care rather than interpersonal conflicts (Sebastião et al., 2024; Sharpe et al., 2023) [38, 39].

Although some literature raises concerns about the limitations of teamwork interventions, the present study reinforces the potential value of structured collaboration in supporting nurses’ vigilance, which is essential for safe and effective patient care in critical care settings (Berrocal Tomé, 2026) [40]. This relevance is particularly important in critical care environments, where nurses face complex patient needs, high workloads, and considerable organizational and occupational demands (Atalla et al., 2025a; Atalla et al., 2025b) [41, 42]. Beyond individual teamwork competencies, successful improvement initiatives in nursing may also depend on organizational transparency, effective leadership, and readiness for change, which can facilitate the implementation and sustainability of quality‐improvement strategies (Atalla et al., 2026; Scortzaru et al. 2024) [43, 44]. Complementing these organizational considerations, adaptability in nursing leadership itself appears to shape how well teams function under evolving conditions. López‐Martín, Zabala Baños, and Dorado Chaparro, 2025 [45] found that nurse managers who adopted flexible, responsive leadership approaches, particularly within hybrid and remote care models, achieved measurable gains in staff engagement and retention. Such findings lend further support to the notion that safe, vigilant practice is not solely a product of frontline collaboration but is also shaped by the wider leadership climate that enables teams to remain cohesive, resilient, and committed to shared safety outcomes, even as care delivery structures continue to change (Miller and Nanavaty, 2026; Yıldırım, Çatal, 2024; Beiboer et al., 2023) [46–48].

## 5. Conclusion

Vigilance scores among critical care nurses were statistically significantly predicted by overall perceived TeamSTEPPS teamwork, which was also strongly correlated with nurses’ vigilance. Overall teamwork accounted for 53.3% of the variance in vigilance ratings in the regression model, indicating a strong correlation. These results emphasize how crucial cooperation skills are to nurses’ vigilance and patient safety in critical care environments. Nevertheless, causal interpretation is constrained by the cross‐sectional design. To find out if enhancing TeamSTEPPS teamwork leads to ensuing increases in nurses’ vigilance, more longitudinal and intervention research is required.

## 6. Implications for Nursing Managers

The development of teamwork skills in critical care teams, such as communication, mutual support, leadership, and situation monitoring, should be a top priority for nursing managers. According to the current research, these teamwork characteristics are positively correlated with nurses’ vigilance and could serve as helpful goals for programs aimed at quality improvement, team building, and education. Effective patient monitoring and patient safety may be facilitated by techniques that promote shared situational awareness, chances for interprofessional communication, and structured collaboration training. However, before assuming causal impacts on nurses’ vigilance or patient outcomes, such tactics should be assessed through longitudinal or intervention research.

## 7. Limitations and Further Research

The descriptive‐correlational nature of the study examining the relationship between TeamSTEPPS teamwork and nurses’ vigilance among critical care nurses means that it captures data at a single point in time. Consequently, it cannot establish causal relationships between TeamSTEPPS training interventions, teamwork dynamics, and nurses’ vigilance. Longitudinal studies are needed to assess how these variables influence one another over time.

Conducting the study in a single setting, such as a specific critical care unit, may limit the generalizability of the findings to other healthcare environments. Results may differ in various hospitals or regions due to disparities in management practices, cultural factors, or organizational structures that impact teamwork and vigilance. The reliance on self‐reported questionnaires (such as those assessing TeamSTEPPS, teamwork, and vigilance) could introduce bias, as respondents may provide socially desirable answers. This can lead to an overestimation or underestimation of the actual effects of teamwork on nurses’ vigilance.

A more diverse sample across multiple critical care settings may yield a broader understanding of these phenomena. This can lead to an overestimation or underestimation of the actual effects of teamwork on nurses’ vigilance. Additionally, the consistency of the association between perceived TeamSTEPPS teamwork and nurses’ vigilance, with the self‐report questionnaires used in measuring both of these variables within the same participants at the same time point, may be prone to common method variance, which may have contributed to an inflation of this observed association between the study variables. Future research should consider including objective measures of vigilance, data from multiple sources, or temporally separated measures of predictor and outcome variables to reduce potential common method variance and increase the validity of the relationships observed.

The categorization of teamwork and vigilance scores should be interpreted cautiously because these categories were not based on validated normative thresholds and may reduce the precision of continuous measurement. Another limitation is that nurses were recruited from 23 critical care units within the same hospital. Consequently, observations may not have been completely independent because nurses working within the same unit could share similar organizational environments, leadership practices, communication patterns, and teamwork experiences. The statistical analyses did not account for potential clustering at the unit level through multilevel modeling or cluster‐adjusted standard errors. Therefore, standard errors may have been underestimated. Future research should employ hierarchical or multilevel analytical approaches that explicitly account for the nested structure of nurses within clinical units.

The use of simple linear regression also limits the interpretation of statistical prediction because the primary model did not adjust for demographic or occupational characteristics that may be associated with both teamwork and vigilance. Although age, years of experience, and educational level were examined separately, the primary regression model was intentionally specified as an unadjusted model to estimate the statistical association between overall TeamSTEPPS teamwork and vigilance. Future studies should consider multivariable and multilevel analytical approaches that simultaneously account for potential individual‐level confounders and the clustering of nurses within critical care units.

Future research should consider employing a longitudinal design to assess the long‐term effects of TeamSTEPPS on nursing outcomes, specifically workplace vigilance. This approach could help clarify potential causal relationships and changes over time. Conducting similar studies across various critical care environments (e.g., urban versus rural hospitals) would enhance the comprehension of how TeamSTEPPS impacts nurses’ vigilance in different contexts. Incorporating qualitative methods, such as interviews or focus groups, could provide richer insights into nurses’ experiences and perceptions regarding teamwork under TeamSTEPPS practices and their influence on vigilance. Additionally, research could investigate the effects of implementing TeamSTEPPS through intervention studies, evaluating outcomes before and after its implementation to determine effectiveness in enhancing nurses’ vigilance. Future studies could also compare the effects of TeamSTEPPS in nursing with other teamwork frameworks to ascertain which approaches yield the highest levels of vigilance among critical care nurses.

## Author Contributions

Conceptualization: Amal Diab Ghanem Atalla, Marwa S. Sorour, Amira M. Ali, Ibrahim Alasqah, and Nada Alqarawi. Methodology: Amal Diab Ghanem Atalla, Marwa S. Sorour, and Amira M. Ali. Investigation: Amal Diab Ghanem Atalla, Marwa S. Sorour, and Amira M. Ali. Formal analysis: Amira M. Ali and Ibrahim Alasqah. Resources: Amal Diab Ghanem Atalla, Marwa S. Sorour, and Nada Alqarawi. Project administration: Ibrahim Alasqah and Nada Alqarawi. Draft‐writing: Amal Diab Ghanem Atalla, Marwa S. Sorour, Amira M. Ali, Ibrahim Alasqah, and Nada Alqarawi. Validation: Amal Diab Ghanem Atalla, Marwa S. Sorour, and Nada Alqarawi.

## Funding

The researchers would like to thank the Deanship of Graduate Studies and Scientific Research at Qassim University (https://www.qu.edu.sa/) for financial support (QU‐APC‐2026).

## Disclosure

All authors read and agreed to the final version of the manuscript.

## Conflicts of Interest

The authors declare no conflicts of interest.

## Supporting Information

Additional supporting information can be found online in the Supporting Information section.

## Supporting information


**Supporting Information 1** The supporting information accompanying this article provides additional methodological details to support the interpretation of the study findings. Supporting Figure 1 presents the participant recruitment and enrollment flow diagram, including eligibility assessment, exclusions, refusals, and the final analytic sample. Supporting Table 1 provides the detailed mapping of the 24 individual critical care units into the six aggregated specialty categories (Cardiac, Psychiatry and Neurology, Pediatric, Neonatal, Medical, and Chest) used in the descriptive and comparative analyses. These supporting information enhance transparency regarding sample selection and unit classification procedures.

**Supporting Information 2** STROBE Statement—Checklist of items that should be included in reports of cross‐sectional studies.

## Data Availability

The datasets developed and analyzed during the current work will be made available by the corresponding author upon reasonable request.
