# Supplementary material for: TeamSTEPPS Teamwork and Nurses’ Vigilance in Critical Care: A Cross‐Sectional Correlational Study
Source: J Nurs Manag. 2026 Sep 29;2026:7441795. doi: 10.1155/jonm/7441795 (PMC13624384; doi:10.1155/jonm/7441795)
Supplement: Supplementary file 2 [file JONM-2026-7441795-s002.docx]

The total number of nurses who worked in the chosen setting was 365

31 nurses recruited for the pilot study

16 nurses refused to participate

12 nurses were excluded as they worked in the hospital for less than 6 months

The final nurses recruited in the study were 306

**Supplementary Figure 1: Participant flow chart**

**Supplementary Table 1. Mapping of Individual Critical Care Units to Aggregated Specialty Categories**

| **Aggregated Category** | **Sub-Unit 1** | **Sub-Unit 2** | **Sub-Unit 3** | **Sub-Unit 4** |
| --- | --- | --- | --- | --- |
| 1- **Cardiac care unit** | Second Unit | Third Unit | Fourth Unit | Intensive Care Unit of Emergency Operation |
| 2- **Psychiatry and Neurology care unit** | Neurosurgery ICU (1) | Toxicity Unit | Systemic Lupus ICU | Hematemesis ICU |
| 3- **Pediatric intensive care unit** | Neurosurgery Pediatric ICU | Maxillofacial and Plastic Surgery ICU | Burn ICU | Ear, Nose, and Throat ICU (ENT) |
| 4- **Neonatal intensive care unit** | Transitional Intensive Care Unit | New Transitional Unit | Hepatic Transitional ICU | Continuous Renal Replacement Therapy |
| 5- **Medical intensive care unit** | Medical Emergency Unit | Critical Cases | Diabetes ICU | Urology ICU |
| 6- **Chest intensive care unit** | Surgical Emergency Unit | Pulmonology ICU | Anesthesia ICU | (unassigned 24th unit) |

N.B: Each unit was assigned to the aggregated specialty category according to the similarity of its clinical services and patient population.
